# *Bartonella clarridgeiae* in Fleas, Tahiti, French Polynesia

**DOI:** 10.3201/eid1709.102063

**Published:** 2011-09

**Authors:** Tahar Kernif, Philippe Parola, Bernard Davoust, Loïc Plaire, Olivier Cabre, Didier Raoult, Jean-Marc Rolain

**Affiliations:** Author affiliations: Université de la Méditerranée, Marseille, France (T. Kernif, P. Parola, D. Raoult, J.-M. Rolain);; Direction Régionale du Service de Santé des Armées de Toulon, Toulon, France (B. Davoust);; Direction Interarmées du Service de Santé des Forces Françaises en Polynésie Française, Papeete, Tahiti (L. Plaire, O. Cabre).

**Keywords:** bacteria, bartonella, Bartonella clarridgeiae, vector-borne infections, zoonoses, molecular detection, fleas, Tahiti, letter

**To the Editor:** *Bartonella* species are small, gram-negative, fastidious, and hemotropic emerging pathogens that cause various human diseases and circulate between a large variety of mammalian and arthropod vectors. More than 30 *Bartonella* species have been isolated from humans as well as from wild and domestic animals worldwide (*1*). *B. clarridgeiae* was suggested to be a minor causative agent of cat-scratch disease (CSD) in humans, however, this suggestion remains controversial. Usually, the agent of CSD is *B. henselae* and its principal reservoir is domestic cats (*Felis catus*) (*1**,**2*). The principal vector of these 2 species is the cat flea (*Ctenocephalides felis*) (*3**,**4*). We report *Bartonella* species in fleas collected from cats and dogs in Tahiti, French Polynesia.

In October 2009, fleas were collected from 1 cat and 9 dogs in Papeete, capital of Tahiti Island, French Polynesia. Fleas collected were kept in 70% ethanol and sent to the military veterinary service in Marseille, France; these fleas were later sent to Unité de Recherche sur les Maladies Infectieuses et Tropicales Emergentes in Marseilles. The fleas were identified phenotypically by using current taxonomic criteria. DNA from fleas and negative control DNA from noninfected laboratory lice were extracted by using a QIAamp Tissue Kit (QIAGEN, Hilden, Germany), as described (*3*).

Flea samples were tested for *Bartonella* spp. DNA by using the 7900 HT Fast Quantitative Real-Time PCR System (Applied Biosystems, Foster City, CA, USA) and primers and Taqman probes specific for the 16S–23S rRNA gene intergenic spacer region as described (*5*). Fleas were considered positive when cycle threshold was <30. All positive fleas at screening were confirmed by using standard *Bartonella* PCR and sequencing of partial internal transcribed spacer gene fragments by using primers URBarto1 and URBarto2, as described (*3*). *B. elizabethae* DNA was used as positive control. DNA sequencing reagents were obtained with BigDye Terminator Cycle Sequencing Ready Reaction Kit (ABI PRISM; Applied Biosystems). The sequences were assembled in Sequencher 4.2 (GeneCodes 2003; www.genecodes.com) and were compared with *Bartonella* sequences available in GenBank.

Overall, 81 fleas were collected from 1 cat (13 fleas) and 9 dogs (68 fleas). All 81 fleas collected were morphologically identified as *C. felis*.

Sample fleas were collected from animals visiting a veterinary clinic for neutering or vaccinations. The overall rate of *Bartonella*-positive fleas by molecular screening with real-time PCR was 7.4% (6/81): 6 fleas from the cat (6/13) and none from a dog (0/68). These positive samples were confirmed after intergenic spacer PCR amplification and sequencing with sequences at 100% identity with *B. clarridgeiae* (GenBank accession no. EU589237).

*B. clarridgeiae* was first isolated from the pet cat of an HIV-positive patient in the United States (*6*). However, *B. clarridgeiae* has never been isolated or detected by molecular methods in humans, and thus its implication as a human pathogen remains controversial. The presence of *B. clarridgeiae* antibodies has been reported in a suspected case of CSD and in a patient with a chest-wall abscess (*4*). However, *B. clarridgeiae* has been detected on fleas from various continents, including Europe, Asia, North America (*1*), Africa; New Zealand, and recently from New Caledonia (*7*).

In France, several studies have reported the molecular detection of *B. clarridgeiae* in the blood of a cat or in cat fleas (*C. felis*), indicating the potential role of fleas as vectors of this organism (*8**,**9*). Prevalence of this bacterium in cat fleas may vary and be as high as 67.9% in cat fleas from France (*3*). Moreover, DNA of *B. henselae* and *B. clarridgeiae* has been reported from cat fleas from New Zealand (*10*). Similarly, co-infection with *B. clarridgeiae* and *B. henselae* has been reported in domestic cats from Europe and Asia (*1*). In our study, all *Bartonella* spp.–positive fleas harbored *B. clarridgeiae* only; all were obtained from cats and none from dogs, similar to findings reported from New Zealand (*10*), although *B. clarridgeiae* has been reported from a flea on a dog in Taiwan (*2*).

Papeete, the capital of Tahiti, is located in the South Pacific Ocean, and remains one of the most visited areas by tourists from all over the world. There are many stray cats and dogs in Tahiti that may be infected with *Bartonella* species and thus serve as a reservoir for these pathogens. Our result confirms the presence of *B. clarridgeiae* in Tahiti and is a warning of the presence of flea-borne bartonellosis and the potential risk of *B. clarridgeiae* or other flea-borne diseases for humans exposed to cat fleas.
